# The natural killer cell dysfunction of aged mice is due to the bone marrow stroma and is not restored by IL-15/IL-15Rα treatment

**DOI:** 10.1111/acel.12291

**Published:** 2014-11-16

**Authors:** Savita Nair, Min Fang, Luis J Sigal

**Affiliations:** Immune Cell Development and Host Defense Program, The Research Institute at Fox Chase Cancer Center333 Cottman Avenue, Philadelphia, PA, 19111, USA

**Keywords:** aging, natural killer cells, immunosenescence, cellular immunology, immune cell development, mice

## Abstract

Immune dysfunctions in the elderly result in increased susceptibility to infectious diseases, cancer, and autoimmune diseases. Natural killer (NK) cells are bone marrow-derived lymphocytes crucial for host defense against several infections and cancer. We have previously shown that compared to young, aged C57BL/6 mice have decreased numbers of mature NK cells in the blood, spleen, and bone marrow, resulting in susceptibility to mousepox, a lethal disease caused by ectromelia virus. Here, we describe further age-related defects in NK cells including reduced proliferation *in vivo*, additional signs of immaturity, and dysregulated expression of activating and inhibitory receptors. Aging also alters the expression of collagen-binding integrins in conventional NK cells and the frequency and phenotype of liver tissue-resident NK cells. We additionally show that the defect in NK maturation is the consequence of deficient maturational cues provided by bone marrow stromal cells. Moreover, we demonstrate that in aged mice, treatment with complexes of the cytokine IL-15 and IL-15Rα induce massive expansion of the NK cells, but most of these NK cells remain immature and are unable to restore resistance to mousepox. The use of rodent model to understand immunosenescence may help the development of treatments to improve the immune fitness of the aged. Our work with NK cells should contribute toward this goal.

## Introduction

The elimination of viruses and resistance to viral diseases requires the coordinated action of both the innate and adaptive immune system. It is well established that the aged are generally more susceptible than the young to viral infections (Leng & Goldstein, 2010; Nikolich-Zugich *et al*., 2012), which has generally been ascribed to intrinsic defects in adaptive T-cell responses. Hence, much of the current efforts in the research of antiviral immunity of the aged are geared to understanding this process. On the other hand, less is known about specific defects in innate immunity of the aged.

NK cells are innate lymphocytes that produce cytokines such as IFN-γ and kill infected and tumor cells. In humans, NK cells are critical for resistance to several viral infections (Orange, 2002; Sun & Lanier, 2011) such as Epstein–Barr virus, human cytomegalovirus, varicella-zoster virus, and herpes simplex virus. In mice, NK cells are essential for resistance to mouse cytomegalovirus (Bukowski *et al*., 1984; Loh *et al*., 2005) and the agent of mousepox, ectromelia virus (ECTV) (Delano & Brownstein, 1995; Fang *et al*., 2008).

NK cells develop mostly in the bone marrow from hematopoietic stem cell precursors that interact with stromal cells to progressively mature and acquire effector functions (Colucci *et al*., 2003). The various stages of NK cell development are characterized by the sequential expression of cell surface molecules. Based on the expression of CD27 and CD11b, NK cells can be subdivided into immature, intermediate-mature, and mature subsets identified as CD27^+^CD11b^−^, CD27^+^CD11b^+^, and CD27^−^CD11b^+^, respectively (Hayakawa & Smyth, 2006).

NK cells express several inhibitory receptors (e.g. Ly49A, Ly49C, Ly49G2, and KLRG1) that maintain NK cell self-tolerance and license their effector functions, and activating receptors (e.g. NK1.1, Ly49D, Ly49H, NKG2D, NKp46) that recognize ligands on target cells to trigger cytolysis and effector cytokine production (Lanier, 2005). The different inhibitory and activating receptors are acquired at defined stages of the NK cell developmental process, and defects in maturation can affect their expression (Kim *et al*., 2002).

In addition to activating and inhibitory receptors, other molecules are differentially expressed during NK cell maturation. For example, surface expression of the chemokine receptor CXCR3 is more prominent in immature than in mature NK cells (Marquardt *et al*., 2010).

The exit of NK cells from the bone marrow and their migration into tissues is crucial for immune surveillance. Integrins are vital in cell–cell and cell–extracellular matrix interactions, cellular differentiation, and cell trafficking (Hood & Cheresh, 2002). A very characteristic integrin of NK cells is CD49b (α2, recognized by mAb DX5), which is usually used to enumerate NK cells in mouse strains such as BALB/c, 129, and DBA/2 (Arase *et al*., 2001) that lack reactivity with the anti-NK1.1 mAb PK136 due to allelic polymorphisms in the *Nkrp1b/c* genes (Giorda *et al*., 1992; Carlyle *et al*., 2006). CD49b is expressed in maturing NK cells right before CD11b (Kim *et al*., 2002). It pairs with the integrin CD29 (β1) to form a collagen receptor with preference for collagens type I and III (Leitinger & Hohenester, 2007). The bone marrow also contains a small population of immature NK cells that are CD49b^−^ (Kim *et al*., 2002). These cells are different from tissue-resident NK (trNK) cells, abundant in liver sinusoids, which are also CD49b^−^ but express the integrin CD49a (α1) (Peng *et al*., 2013; Sojka *et al*., 2014) that also pairs with β1 but to preferentially bind collagen type IV and VI (Leitinger & Hohenester, 2007) Different to immature CD49b^−^ cells in the bone marrow, trNK cells in the liver also express the TNF-related apoptosis-inducing ligand (TRAIL) and are cytotoxic. Moreover, they originate from liver-specific hematopoietic progenitor cells (HPCs) and not from bone marrow precursors (Peng *et al*., 2013; Sojka *et al*., 2014).

Blood cell development is regulated by transcription factors (TF). Tbet and Eomesodermin (Eomes) are TFs of the T-box family that play an important role in NK cell development and maturation (Gordon *et al*., 2010; Daussy *et al*., 2014; Sojka *et al*., 2014). While Tbet and Eomes have redundant functions, the presence of one can downregulate the expression of the other. The liver niche supports early expression of Tbet and represses the expression of Eomes, while the bone marrow environment permits expression of Eomes but not Tbet. Thus, liver-resident trNK cells are Eomes^−^, while conventional NK cells of the bone marrow and spleen are Eomes^+^ (Daussy *et al*., 2014; Sojka *et al*., 2014).

The development and proliferation of NK cells is critically dependent on the cytokine IL-15 which is produced by bone marrow stromal cells (Cui *et al*., 2014). IL-15 is recognized through the IL-15 receptor which is a trimer formed by the soluble IL-15Rα chain together with membrane-bound β and γ chains of the IL-2 receptor. IL-15 and its receptor IL-15Rα are important for NK cell development and survival (Marcais *et al*., 2013). Mice deficient in IL-15 or the IL-15Rα are deficient in NK cells (Lodolce *et al*., 1998; Kennedy *et al*., 2000). Further, treatment of mice with IL-15 or IL-15/IL-15Rα complexes has been shown to induce NK cell proliferation in young mice (Rubinstein *et al*., 2006; Stoklasek *et al*., 2006; Dubois *et al*., 2008; Elpek *et al*., 2010).

We have previously shown that under homeostatic conditions, aged B6 mice have decreased maturation of NK cells in the blood, spleen, lymph nodes, and bone marrow and that this results in impaired migration of NK cells to the draining lymph node (D-LN) and susceptibility to mousepox (Fang *et al*., 2010). The defective maturation of NK cells in aged mice has recently been confirmed by other groups (Chiu *et al*., 2013; Beli *et al*., 2014). In addition to defective maturation, NK cells show loss of NK cell-mediated cytotoxicity and IFN-γ production in both aged humans and mice (Albright & Albright, 1983; Nogusa *et al*., 2008; Fang *et al*., 2010; Hazeldine *et al*., 2012; Solana *et al*., 2012). However, the specific mechanisms that underlie these defects remain unknown.

Here, we show that the NK cells of aged mice have multiple maturation defects including reduced proliferation *in vivo*, multiple signs of immaturity, altered expression of activating and inhibitory receptors, and, intriguingly, altered expression of collagen-binding integrins. We also show that the defect in NK maturation is the consequence of deficient maturational cues provided by bone marrow stromal cells. Moreover, we also show that treatment with complexes of the cytokine IL-15 and IL-15Rα induced massive expansion of the NK cells in aged mice, but most of these NK cells remain immature and are unable to provide resistance to mousepox.

## Results

### Aged mice have increased frequency of immature NK cells with poor proliferative potential

**Figure 1:**
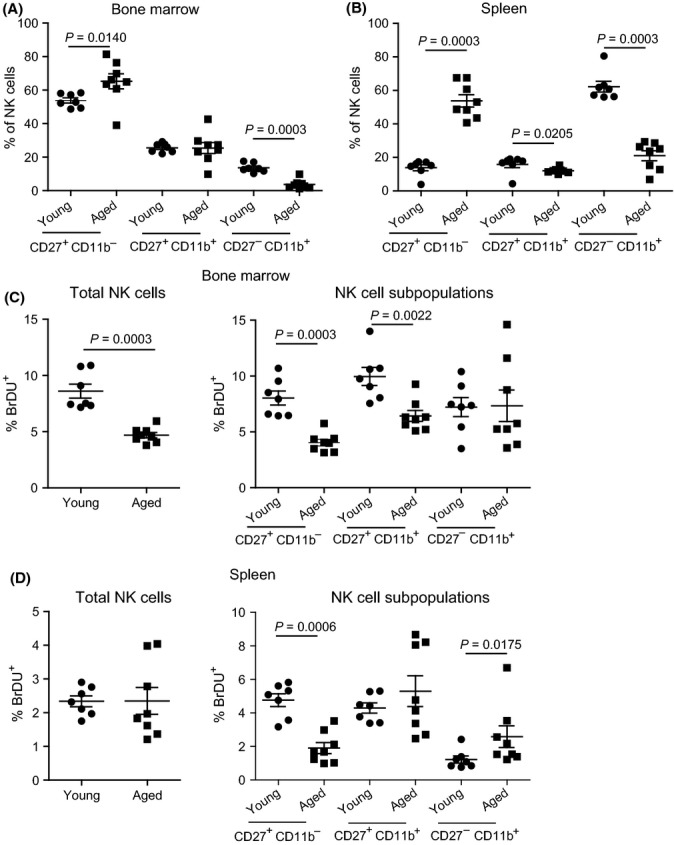
Aged mice have increased frequency of immature NK cells with poor proliferative potential. Young and aged B6 mice were inoculated i.p with 2 mg BrDU and euthanized 16 h later. Bone marrow and spleens were collected, made into single-cell suspensions, and stained for surface NK1.1, CD3e, CD27, CD11b, and intracellular BrdU. Cells were analyzed by flow cytometry using sequential gating on singlets by FSC-A vs. FSC-H, lymphocytes by FSC-H vs. SSC-H, and NK cells (NK1.1^+^ CD3e^−^). (A) Frequency of CD27^+^ CD11b^−^, CD27^+^ CD11b^+^, and CD27^−^ CD11b^+^ NK cells in the bone marrow of young (*n* = 7) or aged (*n* = 8) mice. (B) As A, but in spleen. (C) Frequency of total NK cells (left panel) and CD27^+^ CD11b^−^, CD27^+^ CD11b^+^, and CD27^−^ CD11b^+^ NK cells (right panel) that incorporated BrdU in the bone marrow. (D) As for C but in the spleen. *P*-values were calculated using Mann–Whitney *U* statistical tests. Data correspond to two independent experiments combined. Each data point represents an individual mouse. For all graphs, calculations were performed in the NK1.1^+^ CD3e^−^ gate.

We have previously shown that compared to young, aged B6 mice have lower frequencies of mature CD27^−^ CD11b^+^ NK cells in the bone marrow and spleen (Fang *et al*., 2010). Confirming this, aged mice had a significant increase in the frequency of immature CD27^+^ CD11b^−^ and a significant decrease in mature CD27^−^ CD11b^+^ NK cells in the bone marrow (Fig.1A) and spleen (Fig.1B). We also found that in the bone marrow, total, and more specifically nonterminally differentiated CD27^+^ CD11b^−^ and CD27^+^ CD11b^+^ NK cells proliferated significantly less in aged than in young mice as measured by BrdU incorporation (Fig.1C). In the spleen, there were no significant differences in the proliferative capacity of total NK cells, but there was significantly reduced proliferation of immature CD27^+^ CD11b^−^ and increased (albeit low) proliferation of mature CD27^−^ CD11b^+^ NK cells in aged mice (Fig.1D). Our results are in agreement with a recent study reporting that aged bone marrow has a reduced frequency of proliferating immature NK cells (Beli *et al*., 2014). The increased frequency and reduced proliferation of immature NK cells in the bone marrow of aged mice suggest that full maturation requires proliferation.

### NK cells in aged mice display additional signs of immaturity

**Figure 2:**
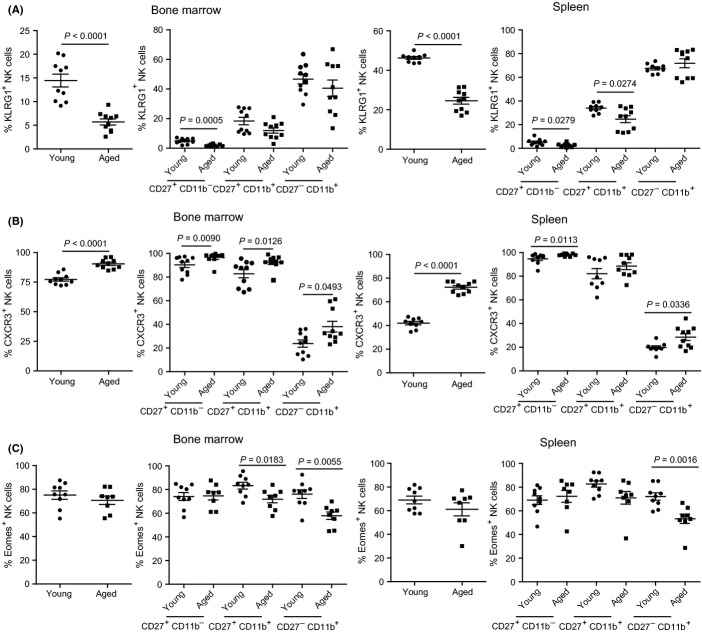
NK cells in aged mice have an immature phenotype. Cells obtained from the bone marrow and spleens of young and aged B6 mice were stained for surface NK1.1, CD3e, CD27, CD11b, KLRG1, CXCR3, and for intracellular Eomes and analyzed by flow cytometry as in Fig.1. (A) Percentages of KLRG1, (B) CXCR3, and (C) Eomes on total NK cells (NK1.1^+^ CD3e^−^) (left panel) and CD27^+^ CD11b^−^, CD27^+^ CD11b^+^, and CD27^−^ CD11b^+^ NK cells (right panel) are displayed. (A–C) *P*-values were calculated using Mann–Whitney *U* statistical tests. Data correspond to two independent experiments combined. Each data point represents an individual mouse. In panels (A&B), *n* = 10 except spleen where the young group had nine mice. In panel C, young (*n* = 9) and aged (*n* = 8) mice.

Our data and that of others (Chiu *et al*., 2013; Beli *et al*., 2014) show NK cell development is hampered in aged mice. To confirm and extend these results, we stained bone marrow and spleens of young and aged B6 mice with mAbs to molecules whose expression is known to be regulated during NK cell maturation. NK cells expressing the inhibitory receptor KLRG1, which is normally expressed in the most mature NK cells (Huntington *et al*., 2007), were significantly reduced on total NK cells in the bone marrow and spleen of aged mice. Additionally, KLRG1 expression was also decreased on immature CD27^+^ CD11b^−^ NK cells in the bone marrow and spleen and in intermediate-mature CD27^+^ CD11b^+^ NK cells in the spleen of aged mice (Fig.2A). CXCR3 is highly expressed on the surface of immature CD27^+^ CD11b^−^ NK cells (Marquardt *et al*., 2010). Consistent with an immature phenotype, total NK cells and NK cell subsets from the bone marrow of aged mice expressed higher frequency of CXCR3. Similarly, CXCR3 expression was elevated on total NK cells and immature CD27^+^ CD11b^−^ and on mature CD27^−^ CD11b^+^ NK cells of spleen from aged mice (Fig.2B).

NK cell maturation and development is regulated by the actions of the TFs Tbet and Eomes (Gordon *et al*., 2010; Daussy *et al*., 2014; Sojka *et al*., 2014). In aged mice, Eomes expression was significantly reduced in mature CD27^−^ CD11b^+^ NK cells in the bone marrow and spleen and in intermediate-mature CD27^+^ CD11b^+^ NK cells in the bone marrow (Fig.2C), On the other hand, as recently shown by others (Daussy *et al*., 2014), the frequency of NK cells expressing the TF Tbet was unchanged (data not shown). Thus, aged mice have fewer mature NK cells in the bone marrow and spleen and further phenotypic characterization revealed that aged mice have altered expression of several NK cell maturation markers.

### Altered expression of activating and inhibitory receptors in the NK cells of aged mice

**Figure 3:**
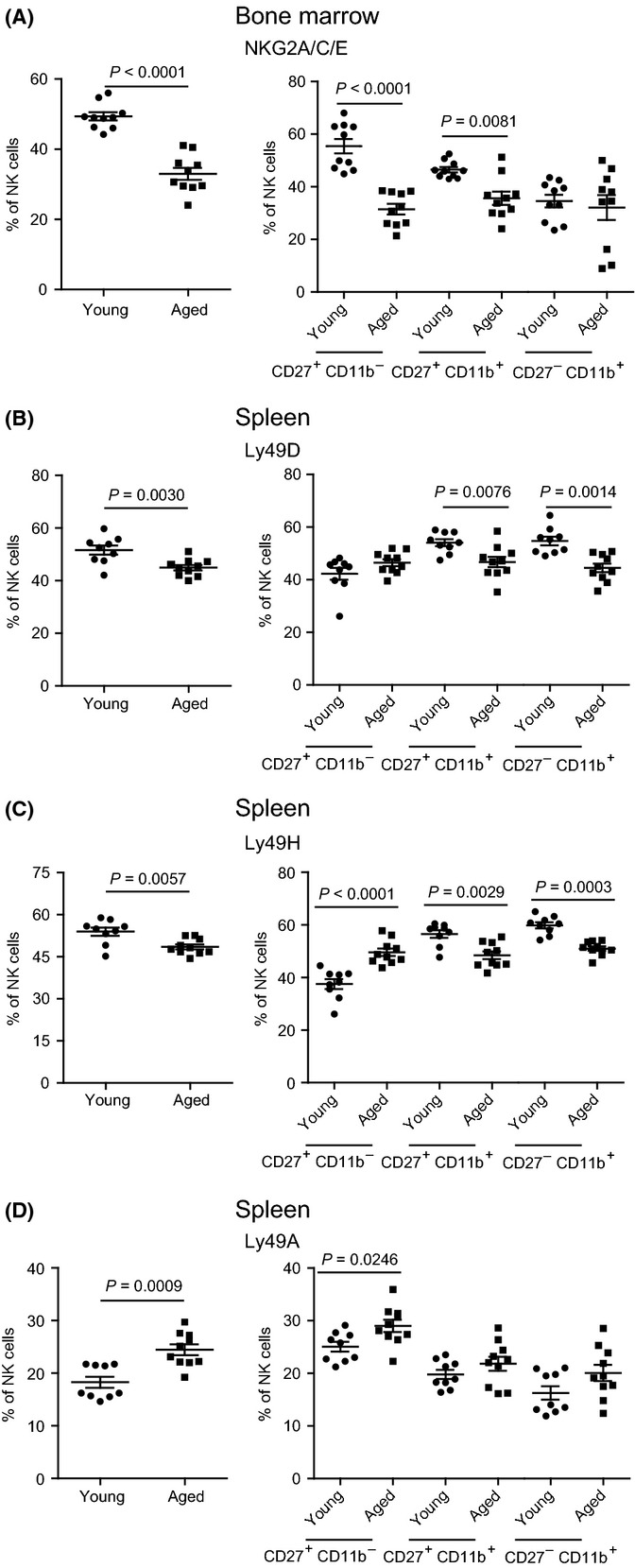
Altered expression of activating and inhibitory receptors in the NK cells of aged mice. Cells were obtained from bone marrow and spleens of young or aged mice and stained for surface expression of NK1.1, CD3e, CD27, CD11b, activating Ly49H and Ly49D, and inhibitory Ly49A and NKG2A (with anti-NKG2A/C/E) and analyzed by flow cytometry as in Fig.1. (A) Frequency of NKG2A/C/E on total NK cells (left panel) and NK cell subsets (right panel) in bone marrow. (B) Frequency of Ly49D on total NK cells (left panel) and NK cells subsets (right panel) in spleen. (C) As in B but for Ly49H. (D) As in B but for Ly49A. *P*-values were calculated using Mann–Whitney *U* statistical tests. Data correspond to two independent experiments combined. Each data point represents an individual mouse. In all the panels, *n* = 10 except spleen where the young group had nine mice.

NK cell licensing and function are dependent on the expression and signaling balance of various activating and inhibitory receptors which are acquired at different times during NK cell maturation (Kim *et al*., 2002). Thus, we compared the expression of various activating and inhibitory receptors in the NK cells from the bone marrow and spleen of aged and young mice. Total, immature CD27^+^ CD11b^−^ and intermediate-mature CD27^+^ CD11b^+^ NK cells in the bone marrow of aged mice had decreased staining with anti-NKG2A/C/E (Fig.3A), which under normal conditions, mostly stains inhibitory NKG2A in NK cells (Vance *et al*., 1999). In the spleen, total NK cells of aged mice had decreased expression of activating Ly49D (Fig.3B) and Ly49H (Fig.3C) and increased expression of inhibitory Ly49A (Fig.3D). When maturational subpopulations were analyzed, the expression of Ly49H was increased in immature CD27^+^ CD49b^−^, Ly49D and Ly49H were reduced in the more mature subsets (Fig.3B and C), and the increase in Ly49A expression was only observed in the immature CD27^+^ CD11b^−^ subset (Fig.3D) of aged mice. Thus, while there were changes in the expression of NK cell activating and inhibitory receptors in the bone marrow and spleen of aged mice, a discernible pattern was not found.

### NK cells in aged mice have altered expression of collagen-binding integrins

**Figure 4:**
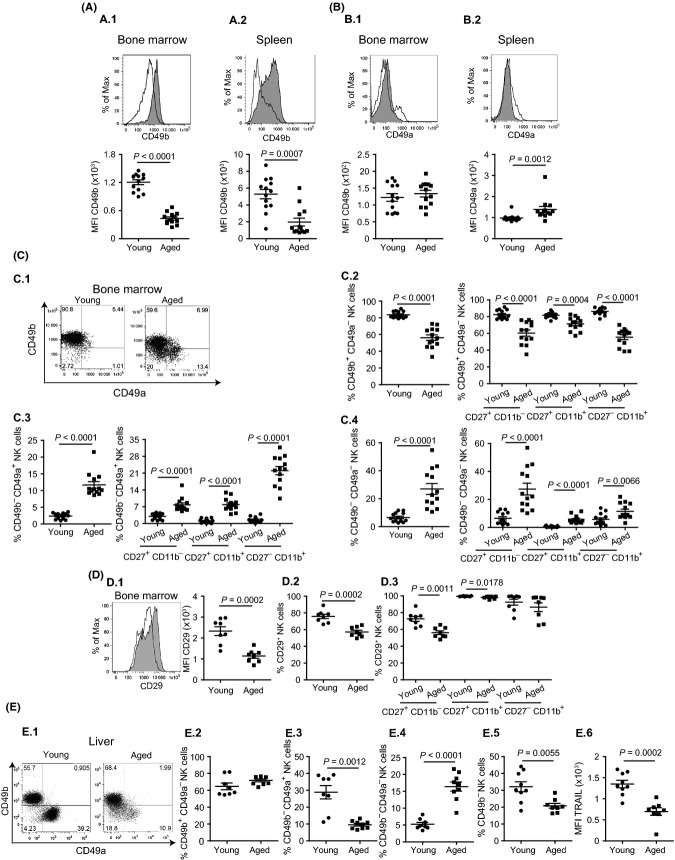
NK cells in aged mice show differential expression of collagen-binding integrins. Cells from bone marrow, spleens, and livers of young or aged mice were stained with mAbs to NK1.1, CD3e, CD27, CD11b, CD49b, CD49a, TRAIL and CD29 (bone marrow and spleen) and analyzed by flow cytometry as in Fig.1. (A) Representative histograms (grayed, young mice, open, aged mice) and summary results showing mean fluorescence intensities (MFI) of CD49b expression in NK cells. (A.1) Bone marrow; (A.2) Spleen. (B) Representative histograms (grayed, young mice, open, aged mice) and summary results showing MFI of CD49a^+^ NK cells in (B.1) Bone marrow; (B.2) Spleen. (C) Differential expression of CD49b and CD49a in the bone marrow. (C.1) Representative flow cytometry plots. (C.2) Frequency of CD49b^+^ CD49a^−^ total NK cells and NK cells at different maturation stages. (C.3) Frequency of CD49b^−^ CD49a^+^ total NK cells and NK cells at different maturation stages. (C.4) Frequency of CD49b^−^ CD49a^−^ total NK cells and NK cells at different maturation stages. (D) Differential expression of CD29 in the bone marrow. (D.1) Representative histograms (grayed, young mice, open, aged mice) and summary and dot plot for CD29 MFI; (D.2) Frequency of CD29^+^ NK cells; and (D.3) Frequency of CD29^+^ NK cells at different maturation stages. (E) Differential expression of CD49b and CD49a in the liver. (E.1) Representative flow cytometry plots. (E.2) Frequency of CD49b^+^ CD49a^−^ total NK cells. (E.3) Frequency of CD49b^−^ CD49a^+^ total NK cells. (E.4) Frequency of CD49b^−^ CD49a^−^ total NK cells. (E.5) Frequency of CD49b^−^ total NK cells. (E.6) MFI of TRAIL in CD49b^−^ total NK cells. *P*-values were calculated using Mann–Whitney U statistical tests. Data are representative of three (A–C) or two (D&E) independent experiments combined. Each data point represents an individual mouse. (A–C) *n* = 13 mice. (D) *n* = 8 mice. (E.1–E.4) young (*n* = 8) and aged (*n* = 9) mice. (E.5–E.6) young (*n* = 9) and aged (*n* = 8) mice.

We have previously shown that NK cells in aged mice fail to migrate to lymph nodes in response to ECTV infection (Fang *et al*., 2010). It is well established that the integrin α2 (CD49b, recognized by mAb DX5) is expressed in conventional NK cells starting at a stage right before the expression of the integrin CD11b (Kim *et al*., 2002) and is usually used as a marker for NK cells. However, its role in the biology of NK cells is unclear. We found that the level of CD49b expression in CD49b^+^ NK cells and the frequency of CD49b^+^ NK cells in the bone marrow and spleen of aged mice were significantly lower than in young mice (Fig.4A).

It has been shown that young B6 mice have a high frequency of trNK cells (CD49b^−^ CD49a^+^, TRAIL^+^) in the livers but not in the bone marrow or spleen (Peng *et al*., 2013; Sojka *et al*., 2014). We found that different to young mice, aged mice had a well-demarked population of NK cells expressing CD49a in the bone marrow (Fig.4B.1) and increased overall expression of CD49a in splenic NK cells (Fig.4B.2). Co-staining for surface CD49b and CD49a molecules (Fig.4C.1) revealed that the frequency of CD49b^+^ CD49a^−^ NK cells in the bone marrow of aged mice was reduced in total NK cells and in NK cells at all maturation stages as defined by CD27 and CD11b expression (Fig.4C.2). Conversely, the frequency of CD49b^−^ CD49a^+^ in total NK cells and in NK cells of different maturational stages was higher in aged mice than in young mice (Fig.4C.3). Notably, the bone marrow of aged mice also had higher frequencies of CD49b^−^ CD49a^−^ cells in total and at different stages of NK cell maturation (Fig.4C.4). However, the CD49b^−^ NK cells in the bone marrow and spleens of young or aged mice were different to the CD49b^−^ TRAIL^+^ Eomes^−^ trNK cells recently described in the liver (Daussy *et al*., 2014; Sojka *et al*., 2014), because they were TRAIL^−^ Eomes^+^ (data not shown).

To form collagen receptors, CD49b and CD49a pair with the integrin β1 (CD29) (Leitinger & Hohenester, 2007). When we tested for CD29 expression, we found that the level and frequency of expression were decreased in total, immature CD27^+^CD11b^−^, and intermediate-mature CD27^+^CD11b^+^ NK cells of aged mice in both the bone marrow (Fig.4D) and spleen (data not shown).

Of interest, when we looked in the livers (Fig.4E), the frequency of conventional CD49b^+^CD49a^−^ NK cells was similar (Fig.4E.1 and E.2), but the frequency of CD49b^−^CD49a^+^ trNK cells was significantly decreased in the livers of aged as compared to young mice (Fig.4E.1 and E.3). In addition, the frequency of a less prominent, still undescribed population of CD49b^−^CD49a^−^ NK cells was significantly increased in aged mice (Fig.4E.1 and E.4). Overall, the frequency of total CD49b^−^ NK cells was decreased in the livers of aged mice (Fig.4E.5), and albeit most of them were TRAIL^+^, the level of surface TRAIL expression at the surface of hepatic CD49^−^ NK cells of aged mice was decreased as compared to those of young mice (Fig.4E.6).

Collectively, Figs3 and 4 show a dysregulated expression of collagen-binding integrin receptors in conventional NK cells of aged mice. Furthermore, they show that the frequency and phenotype of hepatic trNK is affected by aging.

### The defect in the maturation of NK cells in aged mice is due to the aged bone marrow stroma

**Figure 5:**
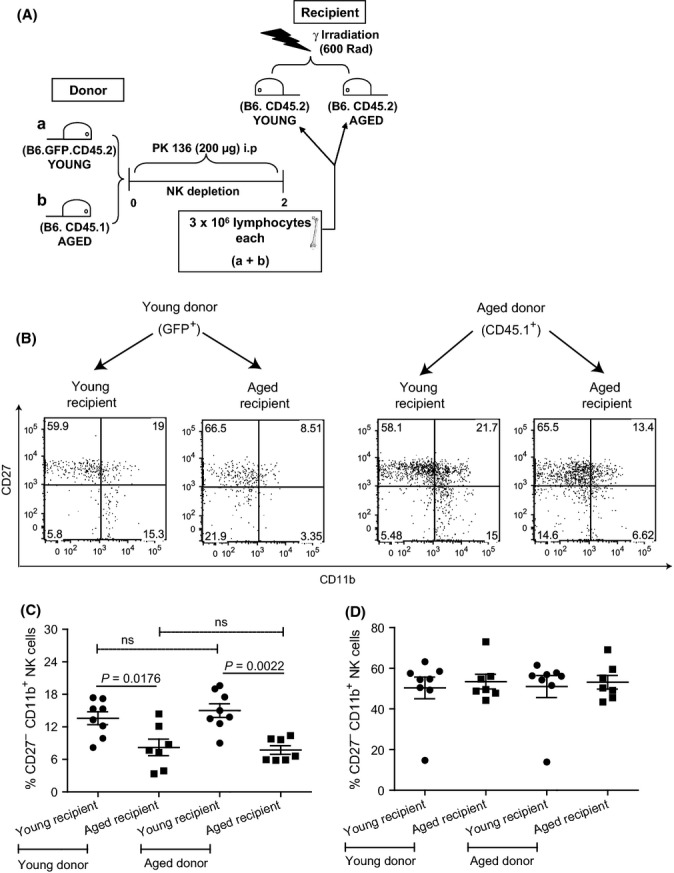
Aged bone marrow stroma is unable to support maturation process. Young B6-GFP mice (CD45.2) and aged B6-CD45.1 mice were depleted of NK cells; their bone marrows collected, mixed 1:1, and used to reconstitute NK cell-depleted-irradiated young or aged B6 (CD45.2) mice. 45 days later, the cells from their bone marrows and spleen were stained with mAbs to NK1.1, CD3e, CD45.1, CD27, and CD11b and analyzed by flow cytometry as in Fig.1. (A) Experimental scheme. (B) Representative flow cytometry plots for bone marrow. (C) Frequency of mature CD27^−^ CD11b^+^ NK cells from young (GFP^+^) or aged (CD45.1^+^) donors in the indicated bone marrows of young or aged chimeras. (D) as for (C) but in the spleen. *P*-values were calculated using Mann–Whitney *U* statistical tests. Data are representative of two independent experiments combined. Each data point represents an individual mouse where young (*n* = 8) and aged (*n* = 7) mice.

NK cells are generated from radio-sensitive hematopoietic precursors mostly in the bone marrow environment that is composed of radio-resistant mesenchymal stromal cells (Colucci *et al*., 2003). To determine whether the NK cell maturation defect in aged mice was due to deficiencies in the hematopoietic precursors or in the stromal cell compartment, we made bone marrow chimeras by sublethally irradiating young and aged B6 mice (CD45.2) and reconstituting them with a 1:1 mixture of bone marrow cells from aged B8-Ly5.2Cr (B6-CD45.1) and young GFP transgenic B6 (B6-GFP, CD45.2) mice that had been depleted of NK cells (Fig.5A). CD45.1^+^ and GFP^+^ NK cells in the bone marrows and spleens of the chimeric mice were analyzed by flow cytometry 45 days after reconstitution. When we compared aged vs. young recipients, aged recipients had higher frequencies of immature CD27^+^ CD11b^−^ and reduced frequencies of mature CD27^−^ CD11b^+^ NK cells from both aged (CD45.1^+^) and young (GFP^+^) donors (Fig.5B and C). NK cells from aged and young donors had similar phenotypes when compared within aged (few mature NK cells) or within young recipients (more mature NK cells) (Fig.5B and C). No differences were observed in the frequencies of mature NK cells in the spleens of young or aged recipients that received hematopoietic cells from either young or aged donors (Fig.5D). Because hematopoietic cells from young mice failed to mature properly in the bone marrow of aged mice, these data indicate a deficiency in the stromal cells of aged mice. On the other hand, because the hematopoietic cells of aged mice produced normal frequency of mature NK cells in the young environment, these data suggest that the hematopoietic precursor cells of aged mice are fully capable of generating mature NK cells in an appropriate environment.

### Treatment of aged mice with IL-15/IL-15Rα complexes induces a massive expansion of immature NK cells that do not restore resistance to mousepox

**Figure 6:**
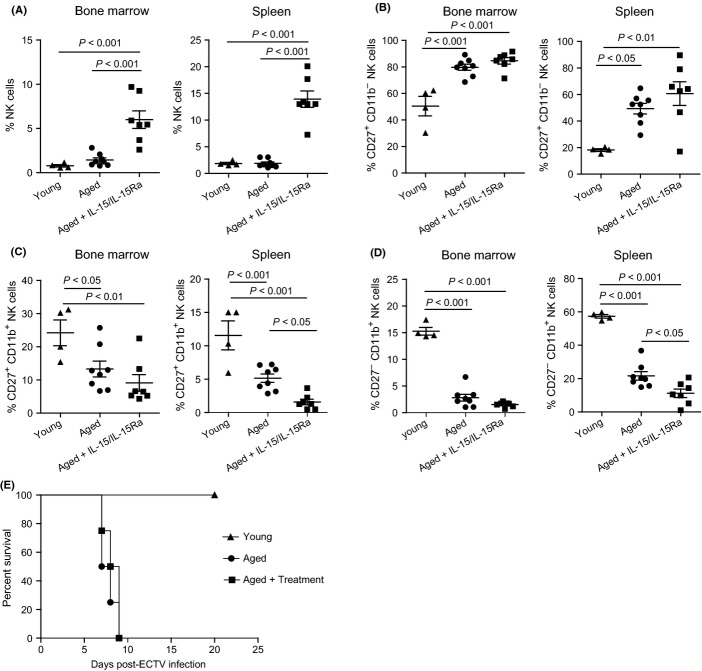
Treatment of aged mice with IL-15 and IL-15Rα complex did not increase frequency of mature NK cells and protect aged mice from mousepox. Aged B6 mice were inoculated i.p. with 0.5 μg of human IL-15 and 3 μg of recombinant murine IL-15Rα in PBS and treated every other day for a period of 2 weeks. Controls were young or aged mice that received PBS. Cells from bone marrow and spleens were stained for NK1.1, CD3e, CD27, and CD11b and analyzed as in Fig.1. (A) Frequency of total NK cells. (B) Frequency of CD27^+^CD11b^−^ NK cells within total NK cells. (C) Frequency of CD27^+^CD11b^+^ NK cells. (D) Frequencies of CD27^−^CD11b^+^ NK cells. *P*-values were calculated using Tukey's test after one-way anova. In (A–D), the data are representative of two independent experiments combined where young (*n* = 4), aged (*n* = 8), and aged + treatment (*n* = 7) mice. (E) Mice were infected with 3000 pfu of ECTV in the footpad and monitored for survival. For humane reasons, the experiment was performed only one time (*n* = 5).

Combined treatment of IL-15 and its receptor IL-15Rα in mice markedly enhances NK proliferation *in vivo* (Rubinstein *et al*., 2006; Stoklasek *et al*., 2006; Dubois *et al*., 2008; Elpek *et al*., 2010). Accordingly, when we treated aged mice with IL-15/IL-15Rα complexes, we found a significant increase in the frequency of total NK cells in the bone marrow and spleen (Fig.6A). The treatment, however, did not affect the frequency of immature CD27^+^ CD11b^−^ NK cells in the bone marrow or spleen (Fig.6B), intermediate-mature CD27^+^ CD11b^+^, or mature CD27^−^ CD11b^+^ NK cells in the bone marrow (Fig.6C and D). Moreover, IL-15/IL-15Rα decreased the frequency of CD27^+^ CD11b^+^ and CD27^−^ CD11b^+^ NK cells in the spleen (Fig.6C and D). Together, these data suggest that in aged mice, IL-15/IL-15Rα increases the number of NK cells by stimulating the production of immature NK cells but does not promote their maturation. We have previously shown that aged B6 mice are susceptible to mousepox due to decreased number and maturation of NK cells (Fang *et al*., 2010). Thus, we tested whether the massive increase in total NK cells induced by IL-15/IL-15Rα complex could improve the resistance of aged B6 mice to mousepox. However, increased resistance did not occur (Fig.6E). This is consistent with the finding that the mature NK cells are those involved in resistance to mousepox (Fang *et al*., 2010, 2011) and show that even very large numbers of immature NK cells cannot protect from mousepox.

## Discussion

While it is now well established that in mice and humans NK cells become dysfunctional with age (Albright & Albright, 1983; Nogusa *et al*., 2008; Fang *et al*., 2010; Hazeldine *et al*., 2012; Solana *et al*., 2012), the whole scope of the dysfunctions and the underlying mechanisms remain unknown. Here, we characterized the impairment of NK cells of aged mice to a greater extent than before, demonstrated that the origin of the defect is in the stroma of the bone marrow, and showed that the maturation and antiviral competence of the NK cells cannot be restored by treating aged mice with IL-15/IL-15Rα.

We have previously reported a decreased number of total NK cells in the blood and spleen and reduced frequencies of CD27^−^ CD11b^+^ mature NK cells in the blood, spleen, lymph nodes, and bone marrow of aged mice. Further, we showed that this defect resulted in the loss of resistance to lethal mousepox (Fang *et al*., 2010). Here, we expanded this finding by demonstrating that immature NK cells in the aged mice proliferate poorly, have additional characteristics of immature cells including decreased KLRG1 and increased CXCR3 expression, and dysregulated expression of Eomes and several inhibitory and activating receptors. Age-associated alterations in the frequency of NK cells, alteration of cell surface receptors, and loss of NK function have been previously observed in both mice and humans (Solana *et al*., 2012). Consistent with our results, others have also recently shown decreased frequencies of KLRG1^+^ (Chiu *et al*., 2013; Beli *et al*., 2014) and increased frequencies of CXCR3^+^ NK cells in aged mice (Beli *et al*., 2014). We also observed an age-associated decrease in Eomes in the more mature NK cell population. Expression of activating and inhibitory receptors was also altered with aging, but the reason and functional consequences of these changes remain to be elucidated.

Intriguingly, we found that aged mice have altered expression of collagen-binding integrins with decreased expression of CD49b and CD29, and increased expression of CD49a in total NK cells and in different NK cell maturation subsets as defined by their expression of CD27 and CD11b in the bone marrow and spleen. Of note, in aged mice, most of the CD49b^−^ NK cells in the spleen are also CD49a^−^, while in the bone marrow, a large fraction of CD49b^−^ cells express the collagen IV/VI receptor CD49a. That NK cells from aged mice have reduced frequencies of CD49b^+^ cells (recognized by mAb DX5) (Arase *et al*., 2001) is surprising because DX5 is frequently used to purify NK cells in many protocols and also to identify NK cells in various mouse strains. Although, the functional role of CD49b in NK cells is not well established, it is known that CD49b^+^ NK cells are the major producers of IFN-γ in the lung of *M. tuberculosis*-infected T cell-deficient mice (Feng *et al*., 2006). More interestingly, it has been recently shown that CD49b and its interaction with collagen fibers regulates the motility and localization of NK cells in lymph nodes during Toxoplasma gondii infection (Coombes *et al*., 2012). As we previously reported, NK cells in aged mice fail to migrate to the D-LN following ECTV infection due to cell intrinsic defects (Fang *et al*., 2010). It is therefore possible that the decreased expression of CD49b in the NK cells of aged mice may have some role in their decreased ability to migrate to D-LNs and protect from mousepox.

Our finding that aged mice have a reduced frequency of hepatic trNK cells (TRAIL^+^ CD49b^−^ CD49a^+^) and an increase in CD49b^−^ CD49a^−^ NK cells is also novel. This finding seems to be unrelated to the increase in CD49b^−^ NK cells in the bone marrow and spleen of aged mice because the latter are TRAIL^−^, and it has recently been shown that TRAIL^+^ liver trNK cells and conventional TRAIL^−^ NK cells have different precursors (Daussy *et al*., 2014; Sojka *et al*., 2014). While the role of hepatic trNK cells remains mostly unexplored, it has recently been shown that they produce memorylike response in NK cell-mediated contact hypersensitivity (Peng *et al*., 2013). Whether they play a role in resistance to viral infections and contribute to the enhanced susceptibility of aged mice to lethal mousepox should be explored further.

Our analysis of mixed bone marrow chimeras showed that the deficiencies of the NK cells in aged mice are not due to intrinsic defects of the hematopoietic precursors but due to an inadequate stroma. A similar observation has just been made by another group (Chiu *et al*., 2013). A characteristic of aging is the decline of lymphopoiesis and an increase in myelopoiesis. The main mesenchymal cell types in the bone marrow that regulate hematopoiesis are osteoblasts and adipocytes. Osteoblasts are essential for lymphopoiesis, while bone marrow adipocytes are known to suppress lymphopoiesis and promote myelopoiesis. Moreover, a deficit in osteoblasts results in decreased numbers of hematopoietic stem cells in the bone marrow. Increased bone marrow adipogenesis and decreased proliferation and maintenance of osteoblasts are characteristics of aging (Chinn *et al*., 2012). It is known that age-associated changes in the bone marrow stroma decrease the differentiation of pro-B into pre-B cells and result in reduced expression of *rag2* and V(D)J recombinase activity in pro-B cells (Labrie *et al*., 2005). Our data show that developmental defects in NK cells of the aged are due to deficiencies in the mesenchymal stromal cells of bone marrow but not due to the hematopoietic stem cells. Notably, the mesenchymal stromal cells are responsible for the production of type I and type IV collagen in the bone marrow (Waterhouse *et al*., 1986). Our data showed that NK cells in aged mice have low expression of α2β1 (CD49b CD29) integrin, receptor for type I collagen with reciprocal increases in expression of α1β1 (CD49a CD29) integrin, and receptor for type IV collagen. Whether these findings are causally related and whether the interaction of developing NK cells with collagen in the bone marrow is required for proper NK cell maturation need to be further explored.

The mesenchymal stromal cells in the bone marrow are also major producers of IL-15 (Cui *et al*., 2014), a cytokine that is essential for the development of NK cells (Marcais *et al*., 2013). It has also been shown that when administered as a complex with IL-15Rα, the half-life and bioavailability of IL-15 *in vivo* are increased, resulting in a massive expansion of the NK cell pool (Rubinstein *et al*., 2006; Stoklasek *et al*., 2006; Dubois *et al*., 2008; Elpek *et al*., 2010). It was therefore plausible to hypothesize that the defect of NK cells in aged mice could arise from defective IL-15 production in the bone marrow and that their numbers and maturation could be increased by IL-15/IL-15Rα treatment. However, while we found that IL-15/IL-15Rα treatment did increase the frequency of NK cells in aged mice significantly, the frequency of mature NK cells was actually reduced. Furthermore, IL-15/IL-15Rα treatment did not restore resistance to mousepox, indicating that the functionality of the NK cells in treated mice was not restored. Consistent with our results, Chiu *et al*. (2013) recently showed that treatment of aged mice with IL-15/IL-15Rα increases the frequency of NK cells as well as the expression of KLRG1 and the cytolytic activity of NK cells, suggesting that IL-15/IL-15Rα treatment could be used therapeutically to restore full functionality to the NK cell compartment of the aged. However, they did not determine the effect of IL-15/IL-15Rα in the frequency of the different NK cell maturation subsets as determined by CD27 and CD11b expression or the NK cell protective function during a pathogenic infection. Our results demonstrating that IL-15/IL-15Rα treatment does not increase the functionally relevant CD27^−^ CD11b^+^ compartment and does not recover resistance to mousepox indicate that this treatment may not be sufficient to restore a fully functional NK cell compartment in the aged and that additional treatments should be explored.

## Experimental procedures

### Mice

The Fox Chase Cancer Center (FCCC) Institutional Animal Care and Use Committee approved the experimental protocols involving animals. Female mice were used for all the experiments. C57BL/6 (CD45.2) mice were purchased from Taconic when they were 6–8 weeks old. Breeders of C57BL/6-Tg(CAG-EGFP)1Osb/J (B6-GFP, CD45.2) mice were purchased from Jackson Laboratories and bred at FCCC. Aged B6 (CD45.2) mice were purchased young from Taconic and aged at FCCC or were purchased as aged from the National Institute of Aging. B6-Ly5.2/Cr (B6-CD45.1) were purchased young from the National Cancer Institute and aged at FCCC. In all experiments, young mice were 6–8 weeks old, while aged mice were 15–18 months old. All purchased mice were rested at least 1 week in the FCCC animal facility before use.

### Viruses and infections

ECTV stocks were produced and titers determined as previously described (Fang *et al*., 2010, 2011). Mice were infected in the left footpad with 25 μl PBS containing 3 × 10^3^ pfu ECTV.

### Cell isolation

Mice were euthanized by cervical dislocation. Single-cell suspensions were prepared from spleen and bone marrow and lysed for red blood cells (RBCs) using Ammonium-Chloride-Potassium (ACK) lysis buffer, and cells were washed with RPMI 1640 supplemented with 5% FCS and later used for flow cytometric analysis. To obtain liver mononuclear cells, anesthetized mice were bled by cardiac puncture, and the liver was isolated, mechanically dissociated with plunger on a 100-μm cell strainer, and filtered through a 70-μm cell strainer. The single-cell suspension was washed once with RPMI media and spun at 524 g for 10 mins at 4 °C. The pellet was resuspended in 40% percoll containing 100 U/ml of heparin, centrifuged for 20 min at 931 g at room temperature, and the RBCs were lysed with ACK, washed with RPMI, and used for flow cytometric analysis.

### Mixed bone marrow chimeras

Young GFP transgenic B6 mice (CD45.2) and aged B6 congenic B6.CD45.1 mice were depleted of NK cells by intraperitoneal administration of 200 μg of PK136 antibody. Two days later, bone marrow cells from the NK-depleted donors were collected, mixed (1:1), and 6 × 10^6^ used to reconstitute young and aged B6 (CD45.2) recipient mice that had been irradiated with 600 Rad. 45 days later, the frequency of mature NK cells (CD27^−^CD11b^+^) among total NK cells was measured in the bone marrow of young or aged recipients.

### BrdU incorporation assay

Mice were injected with 2 mg BrdU i.p. 16 h later, and spleens and bone marrow were removed and made into single-cell suspensions. The cells were stained for cell surface molecules, fixed, and permeabilized using the Cytofix/Cytoperm kit, incubated with DNase at 37 °C for 1 h, stained with anti-BrdU mAb, and analyzed for intracellular BrdU incorporation in total NK cells and subsets.

### IL-15/IL-15Rα treatment

For each treatment, 0.5 μg of recombinant human IL-15 (R&D Systems, Minneapolis, MN, USA) and 3 μg of recombinant murine IL-15Rα Fc chimera (R&D Systems) were mixed and incubated for 30 min at 37 °C. Aged B6 mice were inoculated intraperitoneally with the IL-15/IL-15Rα complex every other day for a period of 2 weeks (Elpek *et al*., 2010).

### Flow cytometry

Flow cytometry was performed as previously described Fang *et al*. (2010). The following antibodies were used: anti-CD3e (clone 145-2C11, Biolegend), anti-CD11b (clone M1/70, Biolegend), anti-CD19 (clone 1D3, BD Biosciences), anti-CD27 (clone LG.3A10, Biolegend), anti-CD29 (clone HMβ1-1, Biolegend San Diego, CA), anti-CD44 (clone IM7, Biolegend), anti-CD45.1 (clone A20, Biolegend), anti-CD45.2 (clone 104, Biolegend), anti-CD49a (clone HMα1, Biolegend), anti-CD49b (clone DX5, Biolegend), anti-CD105 (clone MJ7/18, Biolegend), anti-CD106 (clone 429, Biolegend), anti-BrDU (clone PRB-1, eBioscience), anti-CXCR3 (clone CXCR3-173, Biolegend), anti-Eomes (clone Dan11mag, eBioscience), anti-KLRG1 (clone 2F1/KLRG1, Biolegend), anti-Ly-6A/E (Sca-1) (clone E13-161.7, Biolegend), anti-Ly49A (clone A1/Ly49A, Biolegend), anti-Ly49C/I (clone 5E6, Biolegend), anti-Ly49D (clone 4E5, Biolegend), anti-Ly49G2 (clone LGL-1, eBioscience), anti-Ly49H (clone 3D10, Biolegend), anti-NK1.1 (clone PK136, Biolegend), NKG2A/C/E (clone 20d5, Biolegend), anti-Tbet (clone 4B10, Biolegend), and anti-TRAIL (clone N2B2, Biolegend). At least 500 000 cells were analyzed by flow cytometry at the Fox Chase Cell Sorting Facility using an LSR II system (BD Biosciences).

### Statistical analysis

Statistical analysis was performed with Prism software (Graphpad Software Inc., La Jolla, CA, USA) software. For survival studies, *P*-values were obtained with the log-rank (Mantel-Cox) test. *P*-values were determined using Mann–Whitney test, and when multiple groups had to be compared, we used one-way anova and post-hoc Tukey's for multiple comparisons.
